# A population-based study on the risk of cervical cancer and cervical intraepithelial neoplasia among grand multiparous women in Finland

**DOI:** 10.1038/sj.bjc.6601650

**Published:** 2004-03-02

**Authors:** M Hinkula, E Pukkala, P Kyyrönen, P Laukkanen, P Koskela, J Paavonen, M Lehtinen, A Kauppila

**Affiliations:** 1 Department of Obstetrics and Gynecology, University of Oulu, PL 24, FIN-90029 OYS, Finland; 2 Finnish Cancer Registry, Institute for Statistical and Epidemiological Cancer Research, Helsinki, Finland; 3 National Public Health Institute, Oulu, Finland; 4 Department of Obstetrics and Gynecology, University of Helsinki, Finland; 5 National Public Health Institute, Helsinki, Finland

**Keywords:** cervical cancer, cervical intraepithelial neoplasia, risk factors, parity, human papillomavirus, *Chlamydia trachomatis*

## Abstract

Previous studies suggest that high parity increases the risk of cervical cancer. We studied the risk of cervical cancer (CC) and cervical intraepithelial neoplasia (CIN3) in a Finnish cohort of grand multiparous (GM) women (at least five children) with low prevalence of sexually transmitted infections (STI). The Finnish Cancer Registry data revealed 220 CC and 178 CIN3 cases among 86 978 GM women. Standardised incidence ratios (SIR) were calculated from the numbers of observed and expected cases. Interval analyses by parity, age at first birth and average birth interval were done using multivariate Poisson regression. Seroprevalence of human papillomavirus (HPV) 16 and *Chlamydia trachomatis* was tested among 561 GM women and 5703 women with 2–4 pregnancies. The incidence among GM women was slightly above the national average for squamous cell carcinoma of cervix uteri (SIR 1.21, 95% CI 1.05–1.40) and CIN3 (1.37, 95% CI 1.17–1.58), but lower for adenocarcinoma (SIR 0.77, 95% CI 0.52–1.10). The seroprevalence of HPV16 and *Chlamydia trachomatis* among GM women was lower than in the reference population, except among those women who had their child under age 19. Age under 20 years at first birth increased the risk of CC and CIN3 especially in premenopausal GM women, while increasing parity had no effect. The small relative risks of CC and CIN3 among GM women in our study as compared to studies from other countries can be explained by the exceptionally low prevalence of STIs in Finnish GM women. The observed SIRs between 1.2 and 1.4 should be interpreted to represent increased risk attributable to grand multiparity. The increased incidence of CC and CIN3 among young GM women suggests causal association to HPV 16 and *Chlamydia trachomatis* infections.

Multiparity is believed to be a risk factor for cervical cancer (CC), especially among human papilloma virus (HPV)-positive women (Eluf-Neto *et al*, 1994). The relative risk (RR) of CC among women with five or more births varied from 3.8 (in squamous cell carcinoma) (Munoz *et al*, 2002) to 4.4 (Parazzini *et al*, 1989) in recent studies, compared with nulliparous, or 5.1 compared with nulliparous or primiparous women (Brinton *et al*, 1989).

Human papillomavirus, most notably types HPV16 and 18 (Munoz *et al*, 1994; Lehtinen *et al*, 1996; Dillner *et al*, 1997; Walboomers *et al*, 1999; Wallin *et al*, 1999) and *Chlamydia trachomatis* infections (Koskela *et al*, 2000; Anttila *et al*, 2001) have an important role in the aetiology of CC. Postulated risk factors for CC include, for example, use of oral contraceptives and smoking (La Vecchia *et al*, 1986; Brinton *et al*, 1987b; Schiffman and Brinton, 1995; Schiffman *et al*, 1996; Hakama *et al*, 2000).

We studied the significance of multiparity in the aetiology of cervical cancer of Finnish grand multiparous (GM) women with at least five biological children (Solomons, 1934). Most of the GM women belong to the religious movement of Laestadius within the Lutheran church. High parity is common within this movement, as any kind of contraception is forbidden. Other life habits of the members in this group do not markedly differ from the ordinary Finns. Smoking is permitted, but alcohol consumption is unusual. Since extramarital sexual contacts are unacceptable and rare in this movement, the risk of HPV and *Chlamydia trachomatis* infections of Finnish GM women is presumably low.

## MATERIAL AND METHODS

The computerised files of the Finnish Population Register revealed 86 978 women with at least five children until the end of 1997. The files include links between parents and their children living at the same address in 1974 or later. Follow-up for CC and CIN was done automatically through the files of the national population-based Finnish Cancer Registry with personal identifiers as the key. The cancer registry data also included information about clinical stage and histopathological diagnosis. The CIN group includes patients with histological diagnosis of carcinoma *in situ* or severe dysplasia in the old classification, or CIN grade 3 in the new classification.

The Finnish Maternity Cohort (FMC) of the National Public Health Institute has collected and stored (at −25°C) first trimester serum samples from all (98%) pregnant women in Finland since 1983. Seroprevalences of HPV16 and *Chlamydia trachomatis* were analysed in a stratified random sample of 6264 women with two pregnancies in FMC with standard ELISA methods (Laukkanen *et al*, 2003). These women were linked with the GM cohort. The FMC comprised 561 GM women, whose HPV16 and *Chlamydia trachomatis* seroprevalence was compared with that of women of the same age and period in the rest of sample (*n*=5703).

## STATISTICAL METHODS

The follow-up started from the birth of the fifth child or first of January 1974, whichever was later and ended at emigration, death or 31 of December 1997, whichever overcame first. The number of person years was 1.68 million.

The numbers of observed cases of CC and CIN3 and person-years at risk were counted by 5-year age groups and separately for four parity categories (5, 6, 7 and 8+ children), four categories by the age at first birth (<20, 20–24, 25–29 and 30+ years) and three birth interval categories according to the average interval between the first five deliveries (<2.0, 2.0–3.0 and >3.0 years). Age at follow-up of cancer was categorised into four groups (<40, 40–49, 50–64 and 65+ years).

The expected numbers of cases were calculated by multiplying the number of person-years in each stratum by the corresponding incidence rate in Finland. The standardised incidence ratio (SIR) was calculated by dividing the number of observed cases by the number of expected cases. The 95% confidence intervals (95% CI) for the SIRs were based on the assumption that the number of observed cases presents a Poisson distribution.

Poisson regression modelling of the stratum-specific observed and expected number was used to assess the effects (RR) of all relevant variables simultaneously. The analyses were performed using the SAS statistical software (1997).

## RESULTS

GM women had 220 CC and 178 CIN3 cases, only slightly more than expected (CC; SIR 1.13, 95% CI 0.98–1.29) (CIN3; SIR 1.37, 95% CI 1.17–1.58). The SIR of both CC and CIN3 was smallest among women with at least eight children (Table 1

**Table 1:** Observed (Obs) and expected (Exp) number of cervical intraepithelial neoplasia (CIN3) and cervical cancer (CC) cases and standardised incidence ratios (SIR=Obs/Exp), with 95% confidence intervals (95% CI), among women with at least five children

	**CIN3**				**CC**			
**Variable**	**Obs**	**Exp**	**SIR**	**95% CI**	**Obs**	**Exp**	**SIR**	**95% CI**
*Parity*								
5	92	70	1.32	1.06–1.62	111	98	1.13	0.93–1.36
6	52	34	1.55	1.16–2.03	65	56	1.17	0.90–1.49
7	18	12	1.48	0.88–2.34	24	18	1.32	0.84–1.96
8+	16	15	1.08	0.62–1.76	20	23	0.87	0.53–1.34

*Age at first birth (years)*								
<20	51	29	1.74	1.30–2.29	42	23	1.82	1.31–2.46
20–24	93	71	1.31	1.06–1.61	107	94	1.14	0.94–1.38
25–29	27	25	1.09	0.72–1.58	54	59	0.92	0.69–1.20
30+	7	5	1.33	0.53–2.73	17	20	0.87	0.51–1.39

*Birth interval (years)*								
<2.0	68	42	1.62	1.26–2.05	68	55	1.24	0.97–1.58
2.0–3.0	72	50	1.45	1.13–1.82	78	79	0.99	0.78–1.24
3.0+	38	38	0.99	0.70–1.36	74	62	1.20	0.94–1.50

*Age at follow up (years)*								
<40	38	25	1.51	1.07–2.08	17	7	2.36	1.37–3.77
40–49	62	44	1.42	1.09–1.82	40	29	1.36	0.97–1.86
50–64	59	48	1.23	0.94–1.5	106	100	1.07	0.87–1.29
65+	19	14	1.41	0.85–2.20	57	59	0.96	0.73–1.25

). The younger the age at first birth, the greater was the SIR of CC and CIN3. The SIR of CIN3 was significantly above the national average in women with the average birth interval under 3 years. The excess in incidence of CC was only seen in cancer diagnosed in women under 50 years of age (SIR 1.56, 95% CI 1.18–2.02), 26% of cases.

In all, 190 cases had squamous-cell carcinoma (SCC) (86%) and 30 cases had adenocarsinoma (14%). The incidence of SCC was above (SIR 1.21, 95% CI 1.05–1.40) and that of adenocarcinoma slightly below (SIR 0.77, 95% CI 0.52–1.10) the national average. Increasing parity and long birth interval seemed to decrease the SIR of adenocarsinoma (SIR for 8+ birth, 0.22 and 95% CI 0.01–1.20; SIR for birth interval >3.0 year, 0.41 and 95% CI 0.13–0.95), but the number of cases was small for proper evaluations.

The disease represented clinical (according to Finnish Cancer registry) stage I in 57%, stage II in 11% and stage III in 32% of the 195 cases with a known stage. The Stage II cases had increased SIR (SIR 1.74, 95% CI 1.09–2.64), but in other stages SIRs were about the same as SIR for CC (Stage I, SIR 1.17 and 95% CI 0.97–1.41; Stage III, SIR 1.08 and 95% CI 0.83–1.39).

According to multivariate analysis, the RR for CC in ages under 40 years was 2.30-fold (95% CI 1.27–4.16) compared with cases above 65 years of age, if the reproductive factors were the same (Table 2

**Table 2:** Relative risks (RR) with 95% confidence intervals (95% CI) for cervical intraepithelial neoplasia (CIN3) and cervical cancer (CC) among women with at least five children, by study variables, adjusted for each other

	**CIN3**		**CC**	
**Variable**	**RR**	**95% CI**	**RR**	**95% CI**
*Parity*				
5	1.00	Ref	1.00	Ref
6	1.03	0.72–1.47	1.08	0.78–1.48
7	0.90	0.54–1.53	1.14	0.72–1.82
8+	0.63	0.36–1.11	0.73	0.44–1.21

*Age at first birth (years)*				
<20	1.00	Ref^a^	1.00	Ref
20–24	0.72	0.51–1.02	0.73	0.51–1.06
25–29	0.54	0.33–0.90	0.66	0.42–1.03
30+	0.57	0.24–1.33	0.65	0.35–1.23

*Birth interval (years)*				
<2.0	1.00	Ref^b^	1.00	Ref
2.0–3.0	0.81	0.58–1.15	0.79	0.57–1.11
3.0+	0.52	0.34–0.81	0.91	0.63–1.33

*Age at follow up (years)*				
<40	0.73	0.39–1.37	2.30	1.27–4.18^c^
40–49	0.74	0.40–1.37	1.55	0.95–2.52
50–64	0.74	0.42–1.29	1.25	0.87–1.81
65+	1.00	Ref	1.00	Ref

a Trend *P*=0.02.

b Trend *P*=0.004.

c Trend *P*=0.0003.

). Young age at first birth was associated with increased risk of both CC and CIN3. This phenomenon in CC (Table 3

**Table 3:** Observed number of cervical cancer (CC) cases (Obs) and cervical intraepithelial neoplasia (CIN3) and model-based relative risks (RR) according to study variables with 95% confidence intervals (CI 95%) among GM women, by age at diagnosis

	**CC**						**CIN3**					
	**Age at diagnosis < 50 years**			**Age at diagnosis 50+**			**Age at diagnosis <50 years**			**Age at diagnosis 50+**		
**Variable**	**Obs**	**RR**	**95% CI**	**Obs**	**RR**	**95% CI**	**Obs**	**RR**	**95% CI**	**Obs**	**RR**	**95% CI**
*Parity*												
5	31	1.00	Ref	80	1.00	Ref	55	1.00	Ref	37	1.00	Ref
6	11	0.76	0.37–1.53	54	1.15	0.80–1.64	24	0.97	0.59–1.59	28	1.11	0.67–1.83
7	4	0.68	0.23–1.98	20	1.30	0.78–2.18	12	1.21	0.63–2.31	60	0.62	0.26–1.52
8+	11	1.54	0.70–3.37	9	0.44	0.22–0.91	9	0.80	0.38–1.70	7	0.53	0.23–1.24

*Age at first birth (years)*												
< 20	24	1.00	Ref^a^	18	1.00	Ref	42	1.00	Ref^b^	9	1.00	Ref
20–24	30	0.62	0.36–1.06	77	0.78	0.46–1.30	52	0.68	0.45–1.02	41	1.01	0.49–2.08
25+	3	0.25	0.07–0.83	68	0.71	0.42–1.21	6	0.35	0.15–0.83	28	0.93	0.43–2.02

*Birth interval (years)*												
< 2.0	21	1.00	Ref	47	1.00	Ref	36	1.00	Ref	32	1.00	Ref^c^
2.0–3.0	22	0.99	0.52–1.88	56	0.73	0.49–1.08	43	1.09	0.68–1.73	29	0.58	0.34–0.96
3.0+	14	0.67	0.31–1.47	60	0.97	0.64–1.48	21	0.67	0.37–1.20	17	0.41	0.22–0.78

a Trend *P*=0.01.

b Trend *P*=0.005.

c Trend *P*=0.005.

) or CIN3 (Table 3) was, however, significant only among GM women under 50 years of age. In postmenopausal GM women, an increased CIN3 risk was associated with short birth interval (Table 3).

**Figure 1:**
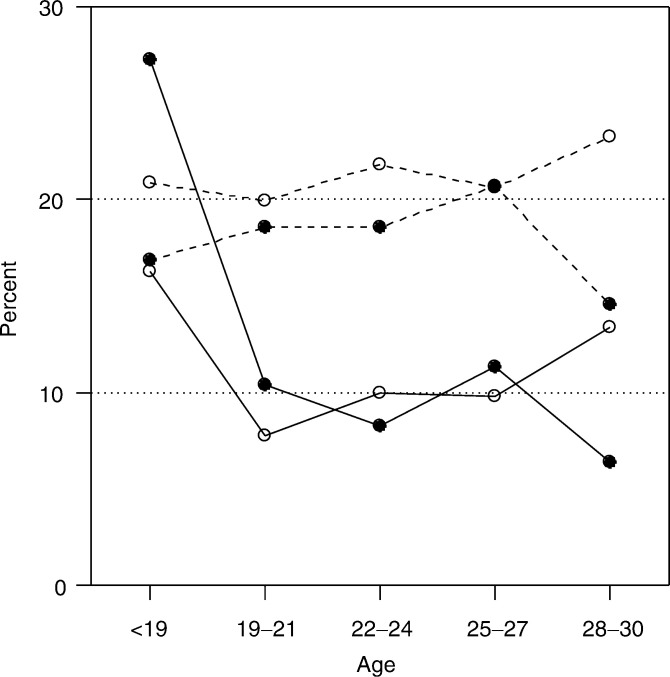
Prevalence of HPV16 (•) and *Chlamydia trachomatis* (○) among GM women (solid line, —), and women with 2–4 pregnancies (dotted lines, - - -), in 1983–1993, by age at pregnancy.

The seroprevalence of HPV16 and *Chlamydia trachomatis* among the 561 GM women included into the FMC sample of 6264 women were 10 and 11%, respectively, which was about half of that in the rest of the sample (Figure 1

). GM women under 19 years of age had during the pregnancy high HPV16 and *Chlamydia trachomatis* seroprevalences (27% and 16%).

## DISCUSSION

We studied the widely accepted hypothesis that multiparity is associated with increased risk for CC and CIN in a national cohort of GM women in Finland. The national population-based databanks of the Finnish Population Register and the Finnish Cancer Registry are virtually complete and the computerised record linkage using personal identifiers as a key is precise.

In this study, the incidence of CC and CIN3 among GM women was slightly higher than that among average female population. In Italy, the RR for CC for 5-paras was 4.4 compared with nulliparous women (Parazzini *et al*, 1989). In the Multicentric IARC study with low- and high-risk countries for 7-paras, the RR of SCC was 3.8 compared with nulliparas and 2.3 compared with women with one or two full-term pregnancies (Munoz *et al*, 2002). In the Latin American countries, the RR was 5.1 for 14+-paras compared with nulli- or primiparous women (Brinton *et al*, 1989). Italian women younger than 45 years with three or more births had an increased risk of 8.1 compared with nulliparous women, and the RR increased with the number of births (Parazzini *et al*, 1998). All these RR estimates are clearly higher than the RR of 1.13 seen in our study. In addition, GM women did not show any trend towards increasing risk of CC or CIN3, with rising number of births from 5 to 8+. The risk estimate was actually lowest in women with 8+ births. Anyway, as our reference group was the entire population with the mean number of 1.5–1.7 births per woman during the study period (www.stat.fi), the RR estimates might have been larger, when compared to nulliparous women.

There are also studies which do not show any role for multiparity in the aetiology of CC or CIN (Rotkin, 1973; Kvale *et al*, 1988; Cuzick *et al*, 1996). In those studies, crude RRs of CC were 0.6–2.3 for women with five or more pregnancies. However, after adjustment for the age at first birth the RR dropped close to 1.0. Similar RRs for CIN have been found in several studies adjusted for age at first birth or number of sexual partners (Parazzini *et al*, 1989, 1992; Jones *et al*, 1990).

The incidence of CC in Finland is low due to the nationwide organised Pap smear screening programmes, conducted since 1965 with 5-year interval among women over 30 years of age (Hakama, 1985). Maternal health care with several pelvic examinations during pregnancy and puerperium (including Pap smears) has been free of charge for over 40 years in Finland with 98% participation rate. These national programmes have promoted the early detection and treatment of CINs, and thus decreased CC incidence in Finnish women. It seems unlikely that these public health programmes have reduced the CC differently in GM women than in the rest of the population. The Pap smear screening and maternal health-care programmes are unlikely to bias our RR estimates. However, these programmes have changed the stage and histology distribution of CC in Finland. The fraction of slow-growing cancers has strongly decreased. Screening also reduces mainly SCC, and therefore the relative proportion of adenocarcinoma increases (Anttila *et al*, 1999). The effect of parity is also dissimilar to different stages or histologies, and this can make it difficult to compare RRs observed in Finland and other countries.

Reproductive factors affect the pathogenesis of SCC and adenocarcinoma in different ways (Munoz *et al*, 2002). The incidence of cervical adenocarcinoma among Finnish GM women was low, a finding that resembles the results of our previous studies on adenocarcinoma of the breast (Hinkula *et al*, 2001) and endometrium (Hinkula *et al*, 2002). Cervical adenocarcinoma may resemble endometrial adenocarcinoma, as regards aetiological association with nulliparity and obesity (Schiffman *et al*, 1996). According to the study by Brinton *et al* (1993), the cervical adenocarcinoma appeared to be less affected by sexual and reproductive factors, whereas in study by Italian, the RR of adenocarcinoma increased with the number of births (Parazzini *et al*, 1988). The epidemiology of cervical adenocarcinoma is still poorly understood (Korhonen, 1980).

There are several pregnancy-induced cervical changes, which may predispose to malignant transformation. Multiparity may increase the risk of CC by maintaining the transformation zone on the ectocervical region. Moreover, the number of squamous metaplastic cells in the transformation zone increases during pregnancy (Munoz *et al*, 2002). In their immature phase of development, the metaplastic cells are most susceptible to HPV infection and possibly later to progression to CC. The metaplastic transformation zone in the ectocervix of a GM woman will repeatedly be exposed to carcinogenetic agents. For this reason, multiparity may intensify the actions of carcinogenic infectious agents (Nair and Pillai, 1992).

Our data did not include any information about the age at first intercourse, smoking or Caesarean sections also thought to be involved in the pathogenesis of CC (Bosch *et al*, 1992; Munoz *et al*, 2002). The GM women are, however, believed to be similar to the average Finnish women with respect to the most general life style factors or manner of delivery. However, in contrast to the reference population, most GM women restrain from using any kind of contraception for religious reasons. Our assumption of the low risk of STIs of the Finnish GM women proved to be true based on the low seropositivity rates to HPV and *Chlamydia trachomatis*, except among those who had children at an average age of less than 19 years. Both of those features are characteristic of our cohort partly for religion reason. There are also other studies with low CC risks in different religious groups as Catholic nuns, the Amish, Mormons and Jews, probably because of a smaller number of sexual partners and lowered infection risk (Schiffman *et al*, 1996).

Infections with oncogenic HPV types are the main causes of CC (Eluf-Neto *et al*, 1994). The pooled data from eight studies indicated that high parity increases the risk of SCC only in HPV-positive women (Munoz *et al*, 2002). There are also conflicting results. Parity may be risk factor of CC rather among HPV-negative than HPV-positive women (Brinton *et al*, 1989). Despite the low prevalence of HPV16- and *Chlamydia trachomatis*-positive women in our cohort compared to the reference population, the SIRs of CC and CIN3 were increased. This finding indicates that multiparity *per se* played a role in the aetiology of these diseases.

GM women who had their first child at young age were an exception to the general tendency; they had a high prevalence of HPV16 and *Chlamydia trachomatis* compared to other age groups and a similar or a slightly higher prevalence than in reference women of the same age. They had an increased risk of CC and CIN3, especially in premenopausal ages. This finding agrees with the results of some (Brinton *et al*, 1987a; Cuzick *et al*, 1990; Bosch *et al*, 1992; La Vecchia *et al*, 1993), but not all previous studies (Parazzini *et al*, 1989, 1998; Munoz *et al*, 1993; Mukherjee *et al*, 1994; Autier *et al*, 1996; Munoz *et al*, 2002).

The importance of age at first birth has been shown to disappear after inclusion parity (Parazzini *et al*, 1989) or the age at first marriage, education and parity (Mukherjee *et al*, 1994) in multiple logistic regression analysis. Theoretically, young age at first birth could be an independent risk factor for CC, because the cervix is most vulnerable at young age, when the risk of sexually transmitted diseases is most prominent. The degree of nuclear atypia increases with the duration of infection (Nair and Pillai, 1992; Schiffman *et al*, 1996).

Short interval between births was associated with an increased risk of CIN3 in postmenopausal GM women. The maintenance of the transformation zone on the ectocervix for a prolonged time increases its susceptibility to the external agents involved in dysplastic lesions (Autier *et al*, 1996). In contrast to a study from India (Mukherjee *et al*, 1994), where birth interval had an independent effect on risk increase of CC, our study, as in a majority of other studies (Munoz *et al*, 2002), failed to reveal such an association for CC.

In conclusion, although the seroprevalence of HPV16 and *Chlamydia trachomatis* in Finnish GM women was small, the incidence of CC and CIN3 was slightly but significantly above the national average. Multiparity seems thus to be an independent risk factor of CC also in a country with effective national programmes for an early detection and treatment of CINs. Young age at first birth also plays a significant role in the aetiology of CC and CIN3, probably in association with STIs.
